# Failure Prediction and Surface Characterization of GFRP Laminates: A Study of Stepwise Loading

**DOI:** 10.3390/polym14204322

**Published:** 2022-10-14

**Authors:** Muhammad Akhsin Muflikhun, Bodo Fiedler

**Affiliations:** 1 Mechanical and Industrial Engineering Department, Gadjah Mada University, Jl. Grafika No. 2, Yogyakarta 55281, Indonesia; 2 Center for Advanced Manufacturing and Structural Application (CAMSE), Gadjah Mada University, Jl. Grafika No. 2, Yogyakarta 55281, Indonesia; 3 Institute of Polymers and Composites, Hamburg University of Technology, Denickestraße 15, 21073 Hamburg, Germany

**Keywords:** failure characteristics, crack propagation, matrix cracking, composite laminates

## Abstract

The present study explores the failure and surface characteristics of Glass Fiber-Reinforced Polymers (GFRP). Stepwise loading was applied in this study to understand the multi-static loading effect on the laminates before final failure. The loading was set three times to reach 10 kN with loading–unloading movement before final load until failure. The results showed that the angle of the GFRP UD laminates’ position significantly impacts the system’s failure. The results were analyzed using theoretical calculation experiment analysis, and then the failure sample was identified using ASTM D3039 standard failure. The laminates with 0° layer on edge ([0/90]_S_ laminates) underwent preliminary failure before final failure. The mechanism of stepwise loading can be used to detect the effect of preliminary failure on the laminates. The [0/90]_S_ laminates are subjected to stress concentration on the edge due to fiber alignment and discontinued fibers in the 0-degree direction. This fiber then fails due to debonding between the fiber and the matrix. The laminates’ strength showed that [90/0]_S_ specimens have an average higher strength with 334.45 MPa than the [0/90]_S_ laminates with 227.8 MPa. For surface roughness, the value of Ra increases more than six times in the 0° direction and three times in the 90° direction. Moreover, shore D hardness showed that the hardness was decreased from 85.6 SD then decreased to 70.4 SD for [0/90]_S_ and 65.9 SD for [90/0]_S_. The matrix debonding, layer delamination and fiber breakage were reported as the failure mode behavior of the laminates.

## 1. Introduction

In recent decades, composites have become the primary materials applied in high-technology systems, including airplane structure, automotive, sports equipment, structure, and electronic components. The first-class properties of composites are that they are lightweight before the metal material, have high resistance to corrosion, have a high tensile strength that can be adjusted by setting the fibers’ angle, exhibit an outstanding fatigue and creep load performance, and have become affordable in recent years [1,2,3]. Among the materials that are used as fibers in composites, glass fiber can be categorized as a low-cost fiber, unlike carbon fiber, with a mediocre–high performance [4,5,6]. Besides the mechanical and physical properties of the fiber that are similar to traditional materials, i.e., steel, composite laminates can also be combined with other materials to reduce weight and increase their performance with a good cost-weight ratio [7,8,9].

Carbon fiber offers excellent mechanical properties and fatigue and creep resistance. Its price, however, is relatively high, and its fracture toughness is rather poor. Glass fiber, on the other hand, has a lower cost and better fracture toughness. Furthermore, its good mechanical and corrosion resistance can broaden its technical applicability. Studies related to the loading performance of GFRP laminates have been reported previously [10,11,12,13,14,15]. The strength prediction of unidirectional (UD) GFRP was evaluated by Kotani et al. [12]. The GFRP laminates were treated via hydrothermal aging before applying tensile loading to determine the system’s strength. The residual strength was predicted, and the degradation of fiber reinforcement and fiber–matrix interface adhesion was observed to cause failure propagation. In another study, Kuboki et al. [13] used an optical microscope to identify failure that generated from a crack and then caused the delamination of the GFRP laminates. Transverse loading was used and generated bending and shear cracks in the matrix laminates. The findings also revealed that the shear cracks grow continuously after being observed using microscopic observation. A study conducting a stress analysis of GFRP laminates was also conducted by Tanabi et al. [15]. A vascularized GFRP was used where the stainless-steel wire was inserted in the middle of the layers. Flexural testing with a three-point bending model was applied in the study to observe the failure behavior. The results showed that the opening channel from the unplugged wire increases the fiber volume ratio and influences the laminates’ strength. The GFRP performance was further studied in the rod system, where the durability and the mechanical properties related to the service life of the specimens were successfully evaluated [16,17].

The failure characteristic of the GFRP laminates became a topic of interest of several scientists. Several analyses and technology adaptions have been introduced to evaluate the failure mechanism. Nair et al. [18] used acoustic emissions (AE) to monitor the damage discrimination in GFRP laminates. Although this can be used to detect preliminary failure in the system, observation is still used. Different failures were reported, such as delamination, fiber breakage, debonding, and matrix cracking. The effect of the flexural loading of the GFRP laminate system was observed by Zhang et al. [19]. The failure is clearly visible from the experimental observations. The results showed that GFRP systems can increase the ultimate moment to 70–323%. In terms of simulation, Bedon and Louter [20] studied the fracture behavior of the GFRP laminates in terms of flexural loading. The study found that simulation analysis can be used to predict failure that is in good agreement with the experimental data. The data, however, need to be clarified with experimental observations to determine the validity of the simulation. Another evaluation technique to observe the failure of GFRP laminates was carried out using a thermography system. An edge notch was applied to both edges of the sample to initiate the stress concentration. The results concluded that the technique can be efficiently used and improved by considering temperature adjustment [21]. Another study by Colombo et al. [22] incorporated simulation analysis using delamination crack propagation using cohesive elements to strengthen the data during the GFRP laminate testing that used thermographic equipment.

Although many studies have been conducted by researchers using advanced technology and implementing the latest techniques, the evaluation of GFRP laminates in fatigue cycles often needs a lot of time and resources to prepare the supporting equipment. Hence, the reliability of the results is questioned if direct observations are not applied. With many papers reporting various techniques, researchers have not yet developed an analysis based on the loading process. One of the loading initiation processes is stepwise loading in GFRP laminates. Stepwise loading can be used to initiate the loading–unloading process of several cycles that take less time than fatigue loading [22]. The FRP mechanical performance and the long-term life prediction over several loading–unloading processes can also be determined and evaluated [23]. The present study aims to determine the failure mechanism and characteristics of GFRP laminates subjected to stepwise loading to fulfill the research gap. A theoretical calculation, loading analysis, and specimen failure evaluation were applied to investigate the effect of loading. A pre-crack specimen based on a stress–strain curve was used, and the standard failure was adopted from ASTM D-3039. This paper contributes to the development of multi-loading in a static test, where pre-crack and early failure were investigated.

## 2. Materials and Methods

### 2.1. Materials

The unidirectional glass fiber used in the present study was available from the market and can be brought from PT Justus Kimiaraya. The epoxy that was used was commercially available with the name Bisphenol-A-Epichlorohydrin epoxy resin mixed with Epoxy Hardener-EPH 555; both were provided by PT Justus Kimiaraya. GFRP was fabricated using the hand lay-up method. The matrix that consists of epoxy mixed with hardener in the ratio of 3:1 was degassed before conducting a manual lay-up on the glass fiber layers to eliminate bubbles that occurred during the mixing process. The material properties of glass fiber and epoxy are shown in Table 1.

### 2.2. The Manufacturing Process and Sample Preparation

**Figure 1:**
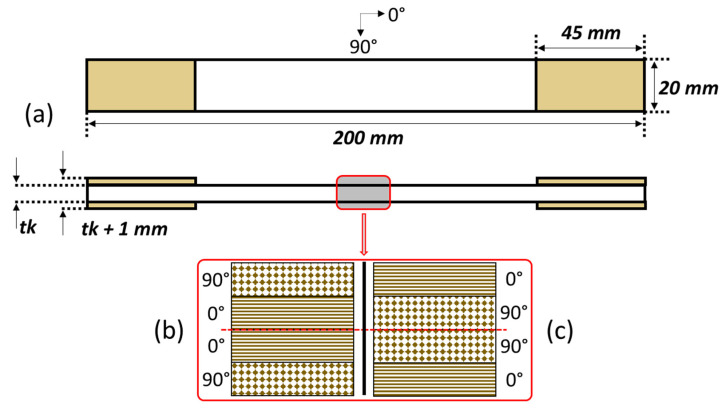
Specimen configuration. (**a**) ASTM D3039 dimension. (**b**) Layer configuration of [90/0]_S_. (**c**) Layer configuration of [0/90]_S_.

The specimen’s geometry is based on the ASTM D3039 standard. Detailed dimensions of the specimen can be seen in Figure 1a, with the thickness of the sample being 3 mm. Two different stacking sequences of GFRP laminates were manufactured with the size 200 × 200 mm—[90/0]_S_ and [0/90]_S_—as shown in Figure 1b,c. Specimens were placed at room temperature for 24 h before being cut to ensure the laminates were fully cured. The specimens were tapped with 0.5 mm aluminum and glued with the epoxy to allow bonding between GFRP and aluminum after the surface of the aluminum and GFRP was sanded using P250 abrasive paper.

### 2.3. Theoretical Calculation

For the theoretical calculation, the properties of GFRP were obtained from a previous study conducted by Cadavid et al. [22]. The calculation used classical lamination theory with the basic ABD matrix. From the calculation of the theory, the experimental results were then compared and examined. The detailed properties of the GFRP used in the present study are shown in Table 2. All the combinations were compared with the present study for further analysis.

### 2.4. Loading Process

**Figure 2:**
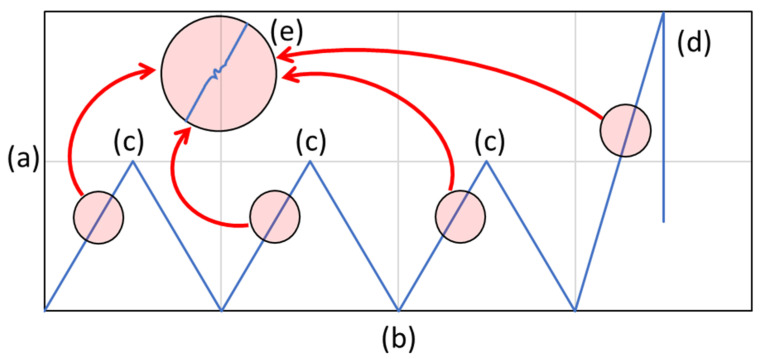
Stepwise loading applied in the present study. (a) Load (N). (b) Time (s). (c) Load limitation (10 kN). (d) Ultimate load (N). (e) Early failure during stepwise loading.

Stepwise loading in the present study was set with the designated load (10 kN) repetition 3 times before the final load until failure, as shown in Figure 2. The loading parameter of the current study used the ASTM D3039 standard, with the loading speed set at 2 mm/min. Using stepwise loading, pre-crack and early specimen failure can be detected. Small inconsistencies in the load–displacement graph can be easily detected, and based on the loading characteristic, stepwise loading can be used to determine the loading far below the final failure when the multi-static load was applied.

### 2.5. Evaluation Process

The present study used different methods to evaluate the laminates before and after being tested. A Zwick Roell UTM Z400 (Shanghai, China) machine with 200 kN capacity was used to determine the load–displacement curves and stress–strain curves for the loading process. The surface roughness Mitutoyo SJ-210 (Kawasaki, Janpan)was used to evaluate the surface before and after testing. For hardness, the Shore D hardness type was incorporated into the sample before and after the test. The optical condition of the surface was checked by using a Dinolite microscope AF4915ZT (New Taipei, Taiwan).

## 3. Results and Discussion

### 3.1. Theoretical Analysis

The theoretical calculation was used to compare and analyze the specimen’s mechanical properties with previous studies. The results could then be used as a general parameter for predicting the different laminates. This theory is based on classical lamination theory (CLT) for fiber composites. Due to their microstructural heterogeneity and behavioral sophistication, the composites have varied tensile, flexural, shear, and torsional mechanical characteristics. Compared to isotropic metal-based materials, describing composites’ constitutive properties appears to be more challenging. Moreover, using theory for the calculation is required to determine the different mechanical properties of the laminates. The ABD matrix showed that Young’s modulus was acquired by using equations and the results will be different by changed the different data. Equations (1) and (2), which are used in CLT, become suitable for calculating mechanical properties such as the specimen modulus.

(1) $$\begin{array}{c} \begin{array}{cc} \left\{\begin{array}{c} N_{x}\\ N_{y}\\ N_{\mathrm{xy}} \end{array}\right\}= & \left[\begin{array}{ccc} A_{11} & A_{12} & A_{16}\\ A_{12} & A_{22} & A_{26}\\ A_{16} & A_{26} & A_{66} \end{array}\right]\left\{\begin{array}{c} \varepsilon_{x}^{0}\\ \varepsilon_{y}^{0}\\ \gamma_{\mathrm{xy}}^{0} \end{array}\right\}+\left[\begin{array}{ccc} B_{11} & B_{12} & B_{16}\\ B_{12} & B_{22} & B_{26}\\ B_{16} & B_{26} & B_{66} \end{array}\right]\left\{\begin{array}{c} \kappa_{x}\\ \kappa_{y}\\ \kappa_{\mathrm{xy}} \end{array}\right\} \end{array}\\ \left\{\begin{array}{c} M_{x}\\ M_{y}\\ M_{\mathrm{xy}} \end{array}\right\}=\left[\begin{array}{ccc} B_{11} & B_{12} & B_{16}\\ B_{12} & B_{22} & B_{26}\\ B_{16} & B_{26} & B_{66} \end{array}\right]\left\{\begin{array}{c} \varepsilon_{x}^{0}\\ \varepsilon_{y}^{0}\\ \gamma_{\mathrm{xy}}^{0} \end{array}\right\}+\left[\begin{array}{ccc} D_{11} & D_{12} & D_{16}\\ D_{12} & D_{22} & D_{26}\\ D_{16} & D_{26} & D_{66} \end{array}\right]\left\{\begin{array}{c} \kappa_{x}\\ \kappa_{y}\\ \kappa_{\mathrm{xy}} \end{array}\right\} \end{array}$$

where

(2) $$\begin{array}{c} A_{\mathrm{ij}}=\sum_{k=1}^{N}{\left({\bar{Q}}_{\mathrm{ij}}\right)}_{k}\left(z_{k}-z_{k-1}\right)\\ B_{\mathrm{ij}}=\frac{1}{2}\sum_{k=1}^{N}{\left({\bar{Q}}_{\mathrm{ij}}\right)}_{k}\left(z_{k}^{2}-z_{k-1}^{2}\right)\\ D_{\mathrm{ij}}=\frac{1}{3}\sum_{k=1}^{N}{\left({\bar{Q}}_{\mathrm{ij}}\right)}_{k}\left(z_{k}^{3}-z_{k-1}^{3}\right) \end{array}$$

**Figure 3:**
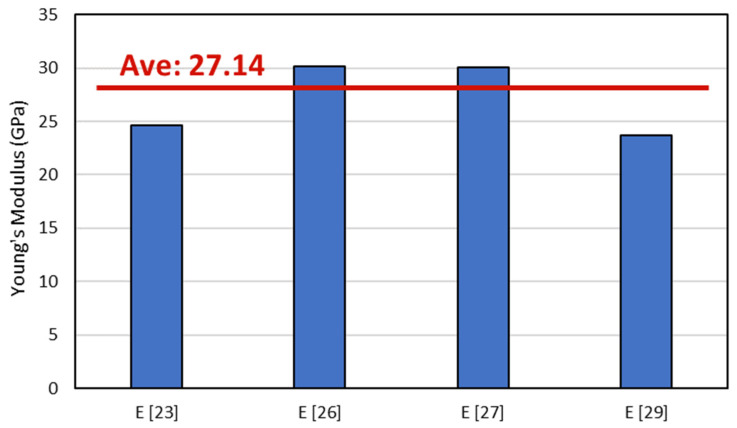
Young’s modulus of different combinations from representative data of previous work.

According to the previous works [29,30], A_ij_ is the extensional stiffnesses, B_ij_ is the bending–extension coupling stiffnesses, and D_ij_ is the bending stiffnesses. Since the laminates are symmetric and tensile loading was used to evaluate the laminates, the value of modulus elasticity can be directly gathered from $A_{\mathrm{ij}}$. A detailed prediction of Young’s modulus can be seen in Figure 3. It is shown that the average Young’s modulus from the previous study was 27.14 GPa, whereas we compared data of the Young’s modulus in the present study with these data. 

### 3.2. Loading Analysis

**Figure 4:**
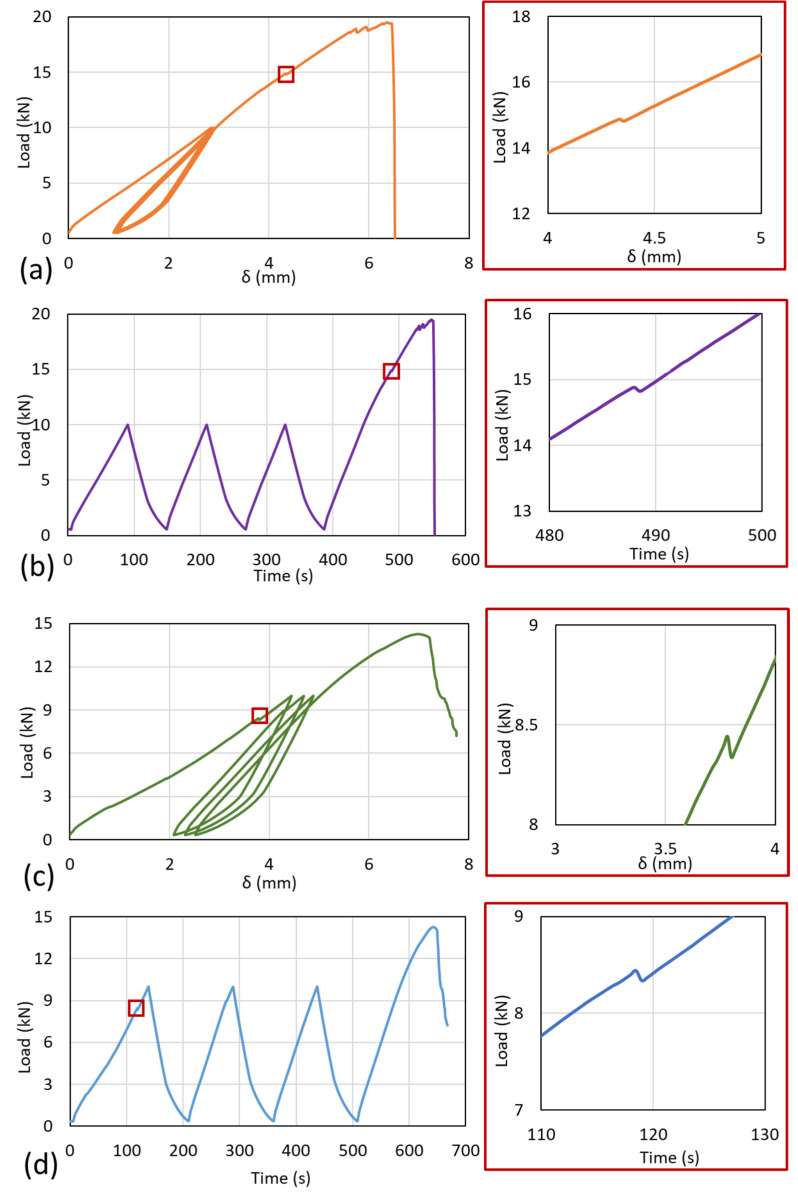
Stepwise loading from the different specimens. The [90/0]_S_ sample with (**a**) load vs. displacement and (**b**) load–time. The [0/90]_S_ sample with (**c**) load vs. displacement and (**d**) load–time.

The loading analysis from the stepwise loading of the representative samples can be seen in Figure 4. The results indicated that both different types of samples were subjected to pre-failure during the stepwise loading. In the [90/0]_S_ samples, pre-failure occurred during the final loading after stepwise loading was applied, as shown in Figure 4a. The stress–strain curve showed that, during the stepwise loading, the elasticity of laminates stood still since the loading showed almost no gap from loadings 1 to 3. The load–time graph with detailed loading can be seen in Figure 4b. It also indicates that right before pre-failure occurred, the load slightly decreased, indicating that failure spots occurred inside the laminates. For the [0/90]_S_ samples, Figure 4c shows that the pre-failure of the specimen occurred during the first stepwise loading. This failure affects the second, third, and final stepwise loadings of the specimen. Although the limit loading was the same as in the [90/0]_S_ samples with 10 kN, as shown in Figure 4d, the stepwise loading process is different and creates hysteresis loading. The hysteresis effect is clearly visible in the [0/90]_S_ sample (Figure 4c,d) because, from the first loading cycle, preliminary failure occurred in the green line with the red circle (Figure 4c). Preliminary failure creates hysteresis that is indicated by the ultimate tensile stress decreasing. These different conditions between the two types of samples indicate that the pre-failure of laminates due to the loading can decrease the strength of the materials even though the laminates have the same sequences.

The crack propagation also occurred in the [90/0]_S_ samples before the final failure, but this phenomenon was not detected in the [0/90]_S_ samples. Crack propagation occurred after the culmination of the failure that occurred in the system laminates. When the pre-failure occurred during the stepwise loading, the system already underwent failure, and the final failure occurred without crack propagation.

### 3.3. Failure Analysis

**Figure 5:**
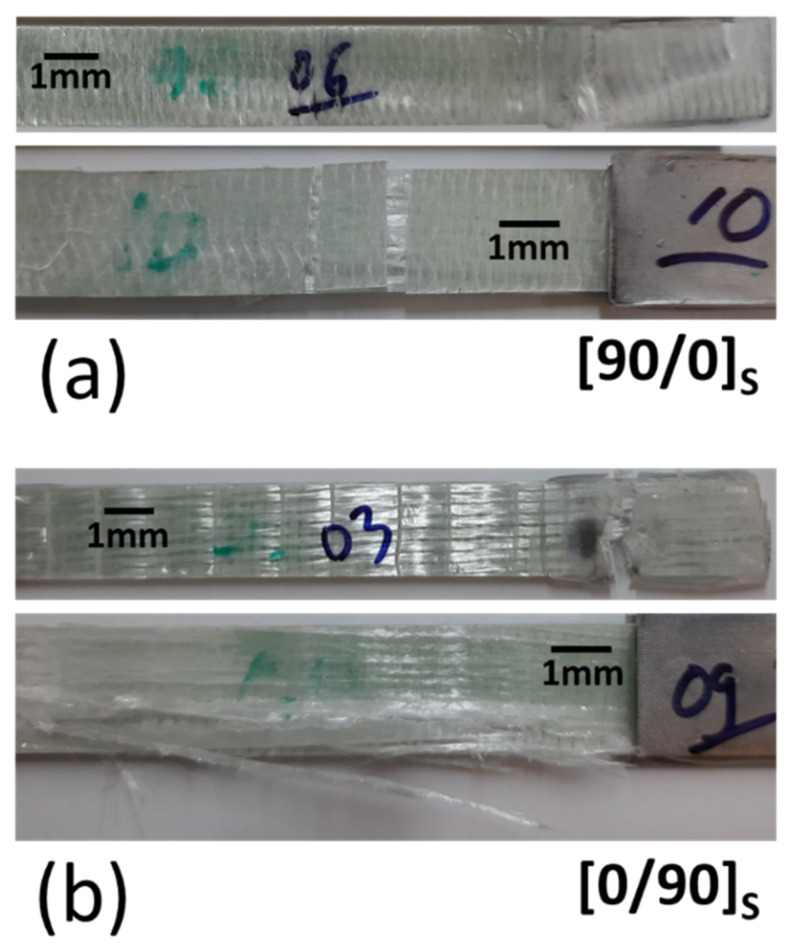
Specimen conditions where the failure was spotted after the test (macro images). (**a**) [90/0]_S_. (**b**) [0/90]_S_.

Macro images of the representative specimen after failure can be seen in Figure 5. The matrix crack clearly occurred on the surface, as seen in Figure 5a. Delamination also occurred in the area under the tab. Matrix cracking occurred after the tensile loading reached the maximum bonding strength of the matrix. The fiber breakages were clearly visible for the [0/90]_S_ sample, as illustrated in Figure 5b. The breakages occurred mainly on the edge of the plate and were distributed in almost all areas. 

**Figure 6:**
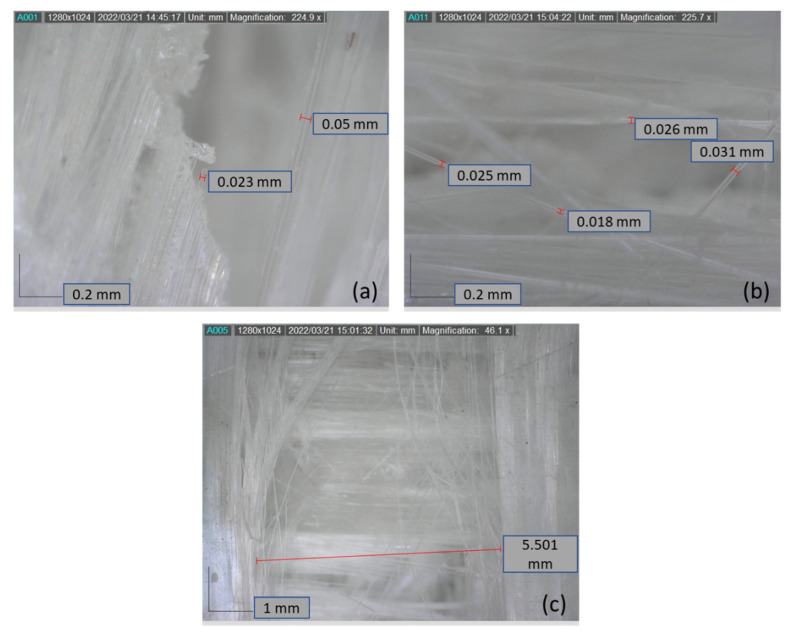
Micro images of failure from different specimens: (**a**) fiber breakage; (**b**) individual fibers’ random angles after failure; (**c**) 90°–0° layers gap after failures.

The specimen was then checked using a microscope to determine the detailed failure mechanism, as shown in Figure 6. Failures were spotted in different forms. Figure 6a showed that the fiber breakage clearly appeared. The fibers and matrix were also stuck together. In a different location, individual fibers were spotted after failure, as shown in Figure 6b. The fiber diameter ranged from 18 µm to 31 µm. These fibers were spotted due to matrix damage, and the fibers were spread at random angles. Another failure due to matrix damage is shown in Figure 6c. It is shown that the damage occurred in form of separated fiber at a 90° angle, making fibers at a 0° angle clearly visible. The gap after failure was around 5.5 mm. 

### 3.4. Surface Roughness

**Figure 7:**
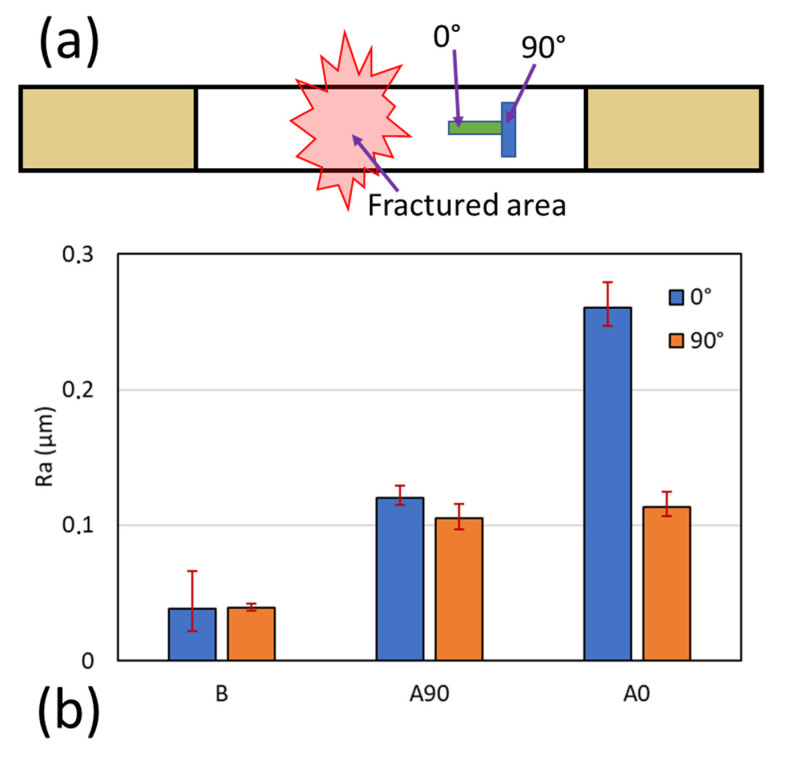
(**a**) The area’s position for gathering surface roughness data. (**b**) Ra results of the specimen. B is the specimen before the test, A90 is the [90/0]_S_ specimen, and A0 is the [0/90]_S_ specimen.

The surface roughness of the specimen was assessed after failure. We checked the representative specimen and checked it three times in all combinations—specimens before and after the test. The summary of the results from the surface roughness assessment can be seen in Figure 7. For the surface roughness evaluation, we used ISO 1997 with the standard profile in λs; the R was 2.5 µm and the length was 0.8 mm, determined using the Gauss model. In Figure 7, the average Ra is presented before the test; the Ra values were found to be 0.038 for the 0° direction and 0.039 for the 90° direction. These results showed that the surface conditions before the test were identical for the 0° direction and 90° direction. After stepwise loading, the roughness of the specimens changed drastically due to the failure that occurred on the surface and in the laminates. The Ra in the 0° direction jumped to 0.26 for the [0/90]_S_ specimen and 0.12 for the [90/0]_S_ specimen. Moreover, the Ra also increased by more than three times in the 90° direction to 0.11 for the [0/90]_S_ specimen and 0.1 for the [90/0]_S_ specimen.

**Figure 8:**
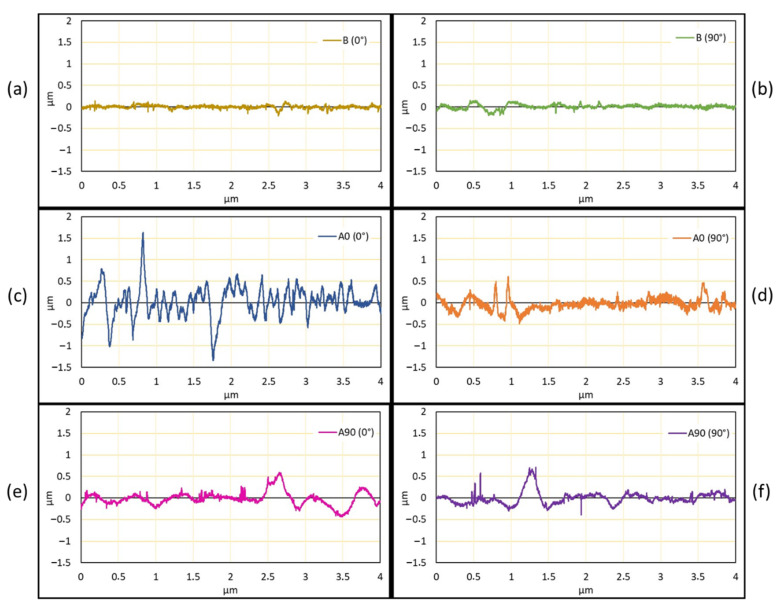
The surface profile of the specimen where the data were captured in the longitudinal directions (0°) and (90°). B is the baseline or sample before loading. A90 is the [90/0]_S_ specimen and A0 is the [0/90]_S_ specimen. (**a**) Surface roughness from 0° direction normal sample. (**b**) Surface roughness from 90° direction normal sample. (**c**) Surface roughness from 0° fiber angle of [0/90]_S_ specimen. (**d**) Surface roughness from 90° fiber angle of [0/90]_S_ specimen. (**e**) Surface roughness from 0° fiber angle of [90/0]_S_ specimen. (**f**) Surface roughness from 90° fiber angle of [90/0]_S_ specimen.

The results from the surface roughness test are shown in Figure 8a–f, which shows the different conditions before and after the final failure. Due to the laminate failure, the surface profile roughened for A0 (0°) and A0 (90°). This was also found for A90 (0°) and A90 (90°). The surface profile of the [0/90]_S_ specimen was more roughened than that of the [90/0]_S_ specimen. This occurred due to the fiber failure condition from the edge. The [0/90]_S_ specimen consists of a 0° layer on the edge. This fiber orientation causes explosive fiber failure (XGM) and, for the [90/0]_S_ specimens, the failure on the edge was Lateral failure (LGM) under the ASTM D3039 definition for fiber failure. The surface profiles also showed that laminate damage was higher for the [0/90]_S_ specimen than the [90/0]_S_ specimen. Moreover, the internal damage showed that the [90/0]_S_ specimen occurred in the middle of layers, where the (0°) layers failed.

### 3.5. Hardness Analysis

**Figure 9:**
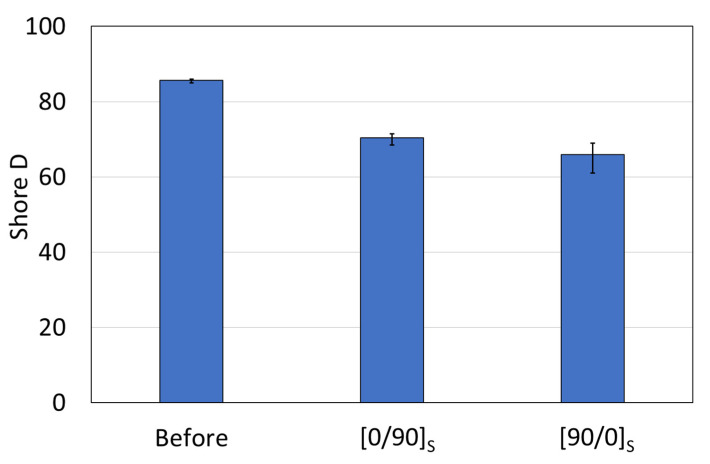
Shore D hardness of the specimen before and after the test.

The hardness test was conducted by using Shore D hardness. At least five points were used to evaluate hardness. The results showed that the hardness values before and after were changed, as shown in Figure 9. The hardness before was recorded with 85.6 SD and then decreased to 70.4 SD for [0/90]_S_ and 65.9 SD for [90/0]_S_. The results indicated that, after failure, the matrix and the fiber were debonded, and the failure caused system rupture, with 0-degree fiber breakage and 90-degree fiber debonding. This result also indicated that the hardness of the specimen can be used to determine the condition of the laminate. If the hardness of the laminate decreases, the failure might occur in the laminates.

### 3.6. Stress Analysis

**Figure 10:**
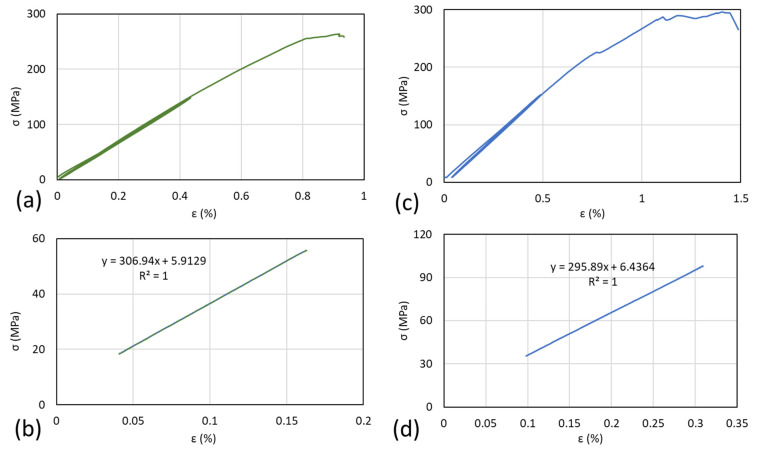
Stress–strain curves of the different samples. (**a**) and (**b**): [0/90]_S_ specimen. (**c**) and (**d**): [90/0]_S_.

The stress analysis of the representative specimen is shown in Figure 10. The moduli of the first load from the [0/90]_S_ specimen and the [90/0]_S_ specimen were 30.7 GPa and 29.6 GPa, respectively. These results agree with previous works [22,29] that reported that the average modulus of the specimen is 30 GPa when using the properties mentioned in that paper. The stress–strain curve of the specimen shows that the [90/0]_S_ specimen has higher ultimate tensile stress and ultimate strain compared with the [0/90]_S_ specimen. It is also showed that both specimens showed pre-cracking that occurred at a different loading stage. For the [0/90]_S_ specimen, the preliminary crack occurred in the first load of stepwise loading, where, for the [90/0]_S_ specimen, the preliminary crack occurred in the last loading when the stress was observed at around 225 MPa. The preliminary crack recorded on the graph indicates that, for the specimen with 0° layers on the edge, the preliminary crack has a higher chance of failure due to the fiber being delaminated. Moreover, the comparison between the experiment and theoretical calculation in Figure 3 showed that the expected results were from the theoretical calculation were correct.

**Figure 11:**
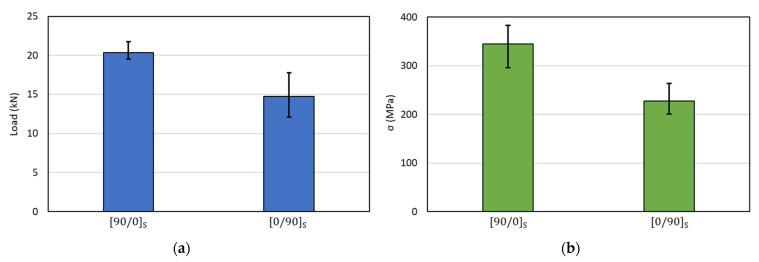
(**a**) The average of maximum load of the specimen and (**b**) strength (maximum stress) of the specimen.

A summary of the maximum loading and ultimate stress of the specimen can be seen in Figure 11. The average load for the [90/0]_S_ specimen was 20.3 kN and for the [0/90]_S_ specimen was 14.7 kN. Moreover, the strength values were 344.45 MPa and 227.8 MPa for the [90/0]_S_ specimen and [0/90]_S_ specimen, respectively. The results indicated that the preliminary failure that occurred during the first, second, or third load can reduce the strength of the specimen drastically. This is comparable to the [90/0]_S_ specimen, where preliminary failure occurred in the final loading.

**Figure 12:**
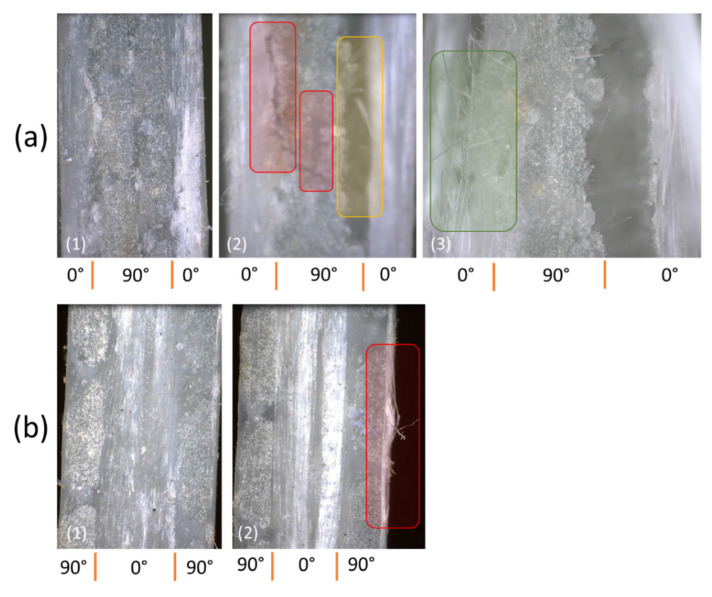
Specimen condition during stepwise loading (1) and after stepwise loading (2)–(3). (**a**) [0/90]_S_. (**b**) [90/0]_S_.

The results from further analysis during the tensile loading of the specimen can be seen in Figure 12. Data from before and after the test for the [0/90]_S_ specimen can be seen in Figure 12a. The specimen condition during loading (1) and after the test (2 and 3) are illustrated. Matrix debonding is clearly shown in the red rectangle, followed by layer delamination between the 90° layer and 0° layer, as shown in the yellow rectangle. As shown in the green rectangle, the breaking of the fiber indicates the specimen’s final failure. Furthermore, the conditions for the [90/0]_S_ specimen before and after the test are shown in Figure 12b. The fiber delamination from the 0° layers was revealed, where the condition of the specimen was not fully damaged like the [0/90]_S_ specimen. These findings showed that, for the specimen that needs to maintain the overall condition, the edge layers should be avoided using the 0° layer. This is because the preliminary crack and failure occurred.

### 3.7. Failure Mechanism

**Figure 13:**
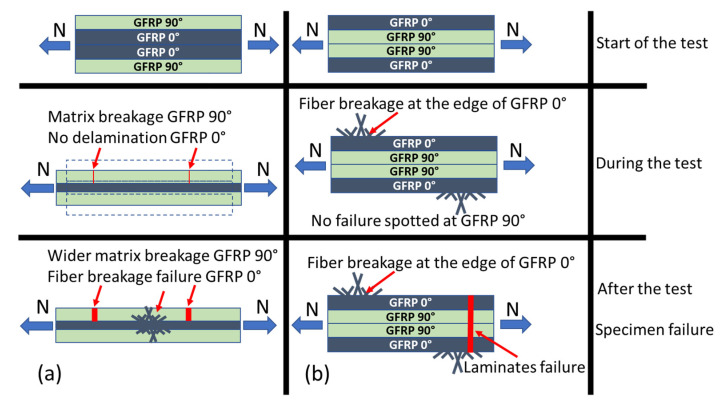
Failure mechanism of the specimen during and after the test was conducted. (**a**) [90/0]_S_ specimen. (**b**) [0/90]_S_ specimen.

This study was able to capture the failure mechanism of the laminates in two different models, as seen in Figure 13. The stepwise loading mechanism that causes the failure of the laminates has been described. For the [90/0]_S_ specimen, as shown in Figure 13a, the loading was mainly charged at the 0° layers due to its superior tensile strength compared to the 90° layers. The specimen also may have a pre-failure crack due to the matrix debonding. This small crack triggered the final failure and caused the gap in the 90° layers to be bigger. The failure mechanism for the [0/90]_S_ specimen is shown in Figure 13b. Since the outer layer was the 0° layer, and there was no matrix cracking after we observed it. Hence, the fiber debonding during stepwise loading occurred and final failure was clearly visible due to fiber breakage; this failure is the main cause of the system failure. Small fiber breakage was reported slightly before the final failure.

## 4. Conclusions

In the present study, stepwise loading was used to explore and determine the failure mechanism of GFRP laminates. Three loading–unloading cycles were carried out with the limit load set at 10 kN before the specimen faced final loading until failure. The laminates with a 0° layer experienced preliminary failure before final failure. The main results are summarized below.

- The strength values of the laminates showed that the [90/0]_S_ specimen has a higher average strength at 334.45 MPa compared to the [0/90]_S_ laminates, at 227.8 MPa.
- The surface roughness test showed that the Ra value increased by more than six times in the 0° direction and three times in the 90° direction. The Ra values increased by 0.038 in the 0° direction and 0.039 in the 90° direction before the test, and 0.26 for the [0/90]_S_ specimen and 0.12 for the [90/0]_S_ specimen after the test.
- The shore D hardness before was recorded with 85.6 then decreased to 70.4 for [0/90]_S_ and 65.9 for [90/0]_S_.
- Matrix debonding, layer delamination, and fiber breakage were reported as the failure mode behaviors of the laminates. The failure of the [90/0]_S_ specimens was caused by matrix debonding and then fiber breakage in the 0° layers. The failure did not occur during stepwise loading, and the debonding occurred before the final failure. Furthermore, the failure of the [0/90]_S_ specimens was mainly due to the fiber breakage in the 0° layers during stepwise loading. This breakage creates system failure and reduces the strength of the laminates.

The present results show that the calculation, surface characteristics, and microscopic analysis strengthened the findings and can be used to conclude that the use of 0° layers for the outer laminates is not recommended in the fiber laminate system.

## Figures and Tables

**Table 1:** Properties of glass fiber and epoxy.

Glass Fiber Properties	Value	Epoxy Properties	Value
Density—*bulk* (g/cm^3^)	2.54–2.62	Viscosity at 25 °C (mPa·s)	13.000 ± 2.000
Coefficient of Thermal Expansion—*bulk* (m/m/K)	4.9–6.0 × 10^–6^	Epoxy number (%)	22.7 ± 0.6
Specific Heat—*bulk* (kJ/kg K)	0.8	Epoxy equivalent (g/equiv.)	189 ± 5
Melting Temp. (°C)	830–916	Epoxy value (equiv./100 g)	0.53 ± 0.01
Thermal conductivity—*bulk* (W/m K)	1	Total chlorine content (%)	<0.2
*Refractive index*—*bulk*	1.547–1.560	Hydrolyzable chlorine content (%)	<0.05
Tensile strength—*filament* (GPa)	3.1–3.8	Colour according to the gardner scale	<1
Modulus—*filament* (GPa)	76–81	Density at 25 °C (g/cm³)	1.17 ± 0.01
*Elongation at break*—*filament* (%)	4.5–4.9	Refractive index at 25 °C	1.572 ± 0.003
* Poisson’s ratio *	0.18	Volatile content at 3 h, 140 °C (%)	<0.2
Dielectric strength—*bulk* (kV/mm)	10.3	Vapour pressure at 80 °C (mbar)	<0.1
Resistivity volume—*bulk* (ohm cm)	1015	Flashpoint according to DIN 51584 (°C)	>250

**Table 2:** Properties of GFRP.

Parameter	GFRP Values							
	[22]	[24]	[25]	[26]	[27]	[28]	[29]	Unit
Longitudinal Modulus E_11_	36	40	43	48	48	64.1	36.9	GPa
Transverse Modulus E_12_	13	8	13	12	12	-	10	GPa
Poisson’s ratio ν_12_	0.22	0.25	-	-	0.19	-	0.32	-
Shear Modulus G_12_	4.3	4	-	6	6	-	3.3	GPa
Shear Strength	48.7	40	-	25	12.5	-	54.5	MPa
Tensile Strength in Fiber Direction	626	1000	-	1200	550	1700	820	MPa
Tensile Strength in Transverse Direction	180	30	-	59	30	-	80.6	MPa
Compressive Strength in Fiber Direction	230	600	-	800	400	-	500	MPa
Compressive Strength in Transverse Direction	180	110	-	128	64	-	322	MPa
Volume Fraction	61	-	51	-	-	81	-	-

## Data Availability

The data supporting the findings of this manuscript are available from the corresponding authors upon reasonable request.
